# Where Have All the Rodents Gone? The Effects of Attrition in Experimental Research on Cancer and Stroke

**DOI:** 10.1371/journal.pbio.1002331

**Published:** 2016-01-04

**Authors:** Constance Holman, Sophie K. Piper, Ulrike Grittner, Andreas Antonios Diamantaras, Jonathan Kimmelman, Bob Siegerink, Ulrich Dirnagl

**Affiliations:** 1 Medical Neurosciences Program, Charité Universitätsmedizin Berlin, Berlin, Germany; 2 NeuroCure Clinical Research Center, Charité Universitätsmedizin Berlin, Berlin, Germany; 3 Center for Stroke Research, Charité Universitätsmedizin Berlin, Berlin, Germany; 4 Department for Biostatistics and Clinical Epidemiology, Charité Universitätsmedizin Berlin, Berlin, Germany; 5 Biomedical Ethics Unit, McGill University, Montréal, Canada; 6 Department of Experimental Neurology, Charité Universitätsmedizin Berlin, Berlin, Germany; 7 German Center for Neurodegenerative Diseases (DZNE), Berlin, Germany; 8 Berlin Institute of Health, Berlin, Germany

## Abstract

Given small sample sizes, loss of animals in preclinical experiments can dramatically alter results. However, effects of attrition on distortion of results are unknown. We used a simulation study to analyze the effects of random and biased attrition. As expected, random loss of samples decreased statistical power, but biased removal, including that of outliers, dramatically increased probability of false positive results. Next, we performed a meta-analysis of animal reporting and attrition in stroke and cancer. Most papers did not adequately report attrition, and extrapolating from the results of the simulation data, we suggest that their effect sizes were likely overestimated.

## Abstract 2

Using a combination of simulation and meta-analysis of stroke and cancer studies, this article highlights the potentially seriously misleading consequences of failing to report the loss of animals in preclinical studies.

*Where have all the rodents gone*?

*Ooh ooh*, *ooh ooh*, *ooh*

*To non-random attrition*, *every one*

*When will they ever learn*?

—with apologies to Pete Seeger, 1955

## Introduction

Research systems worldwide spend billions of dollars every year on developing new drugs [1], yet failure to translate laboratory findings into clinical applications has driven many to question the robustness and predictive value of preclinical research [2,3]. Much of this criticism centers on selection of animal models, internal validity [4,5], statistical power [6–8], reporting, and publication bias [3].

An essential element of the reporting of any preclinical study is the number of samples. These numbers are essential for assessing the statistical power and robustness of results, as well as for including the studies in systematic reviews [9,10]. If done properly, the reporting of animal numbers provides a full account of all animals lost during the experiment. Attrition not only diminishes statistical power but may also represent a nexus for other forms as bias. For example, non-blinded allocation or outcome assessment allows unwanted data to be identified and excluded via reporting bias. Furthermore, in some studies, attrition from the treatment group may be indicative of side effects or toxicity of new treatments. Unreported loss of these animals, therefore, is a potentially harmful form of selection bias.

In clinical research, several meta-analyses show that patient attrition can introduce a form of selection bias that favors positive outcomes [11–13]. To understand the effects of this bias, full disclosure of missing data is needed. Reporting standards, such as the Consolidated Standards of Reporting Trials (CONSORT) and Strengthening the Reporting of Observational Studies in Epidemiology (STROBE) guidelines, require reporting of all dropouts over the course of a study [14,15]. While there are many ongoing attempts to align preclinical research with clinical reporting standards [16–18], compliance with these guidelines is poor [19]. Despite the probable effect of attrition on power in small animal studies [20], its extent and the consequences of attrition in animal research have not, to our knowledge, been studied.

In this study, we set out to demonstrate the consequences and prevalence of attrition in preclinical research. First, we used simulated data and compared the results with and without animal loss. We focused on two kinds of attrition: random loss and biased removal. Here, we investigated the potential impact of animal attrition on detection of clinical promise by examining the probability of false positives and negatives and whether effect size became inflated. Second, we used meta-analytic methods on a sample of preclinical stroke and cancer studies to assess the prevalence of attrition reporting and to determine whether attrition was associated with reported effect size.

## Results

### Simulation Studies

**Fig 1:**
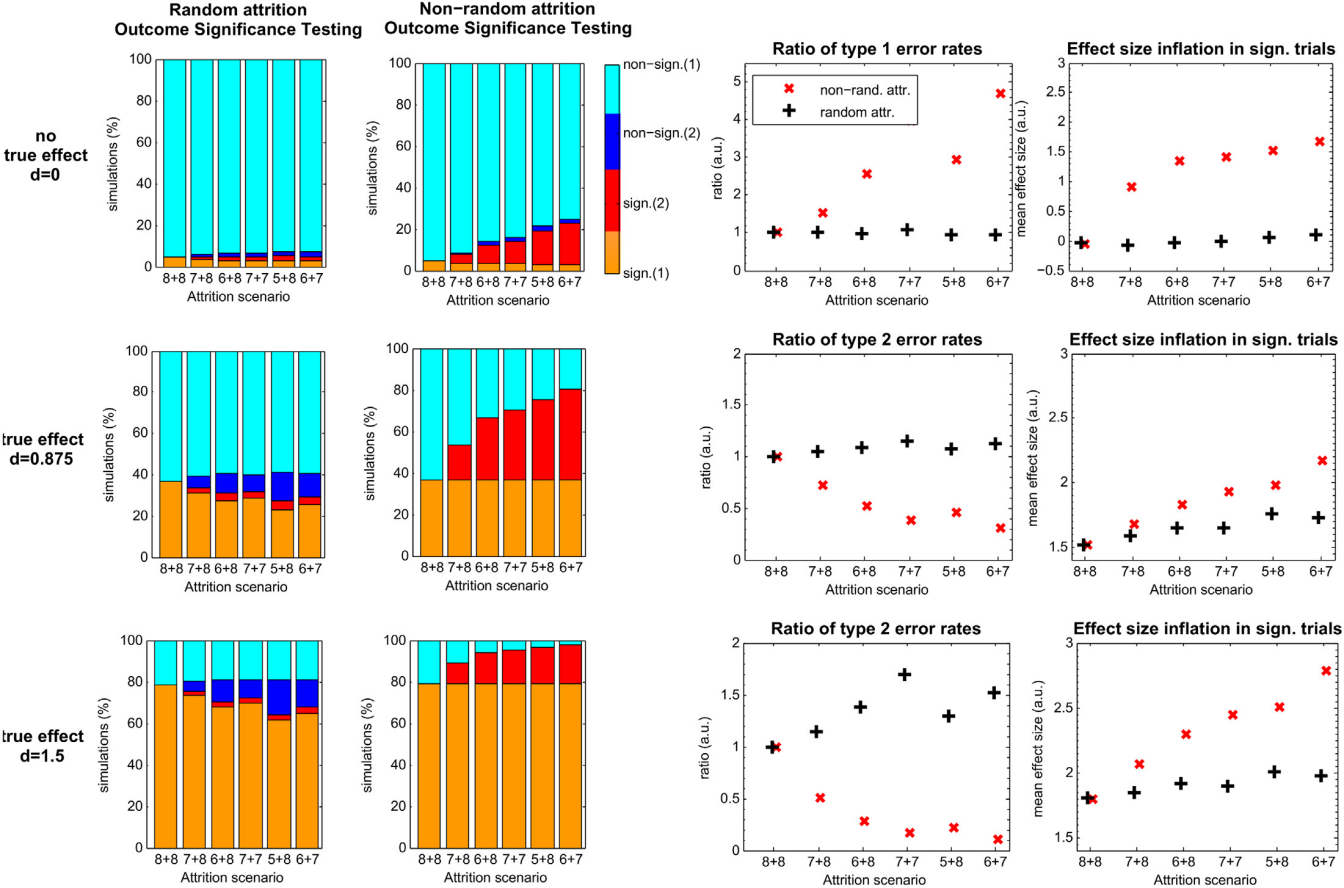
Simulation results for random and non-random attrition. Column 1 + 2: rows represent the results of a different effect size (Cohen’s d) scenario, indicated left. The level of attrition in either treatment group is stated (left to right) as “8 + 8” without attrition and “7 + 8” to “6 + 7” with the total number of missing animals increasing from one to three. Column 1 + 2: probability of positive trials after random loss (first column) or non-random attrition of extremes that are not in favor of the effect (second column) for different effect sizes (rows 1–3). Colors represent the proportion of trials out of 10,000 simulations that are significant (1) independent of attrition (orange) or significant (2) only in the case of attrition (red), non-significant (1) independent of attrition (cyan), or non-significant (2) only in the case of attrition (dark blue). Column 3: ratio of type 1 error rates (falsely accepting the alternative (H_1_) hypothesis if there is no true effect, first row) or type 2 error rates (falsely failing to reject the null hypothesis if there is a true effect, second and third row), respectively, for different levels of attrition relative to the rates acquired with the full sample (“8 + 8”). Ratios for random attrition are colored in black, and ratios for non-random attrition are colored in red, in arbitrary units (a.u.). Fourth column: effect size estimated from positive trials only. Mean estimated effect sizes are displayed in black (+) for random attrition and in red (×) for non-random attrition.

**Fig 2:**
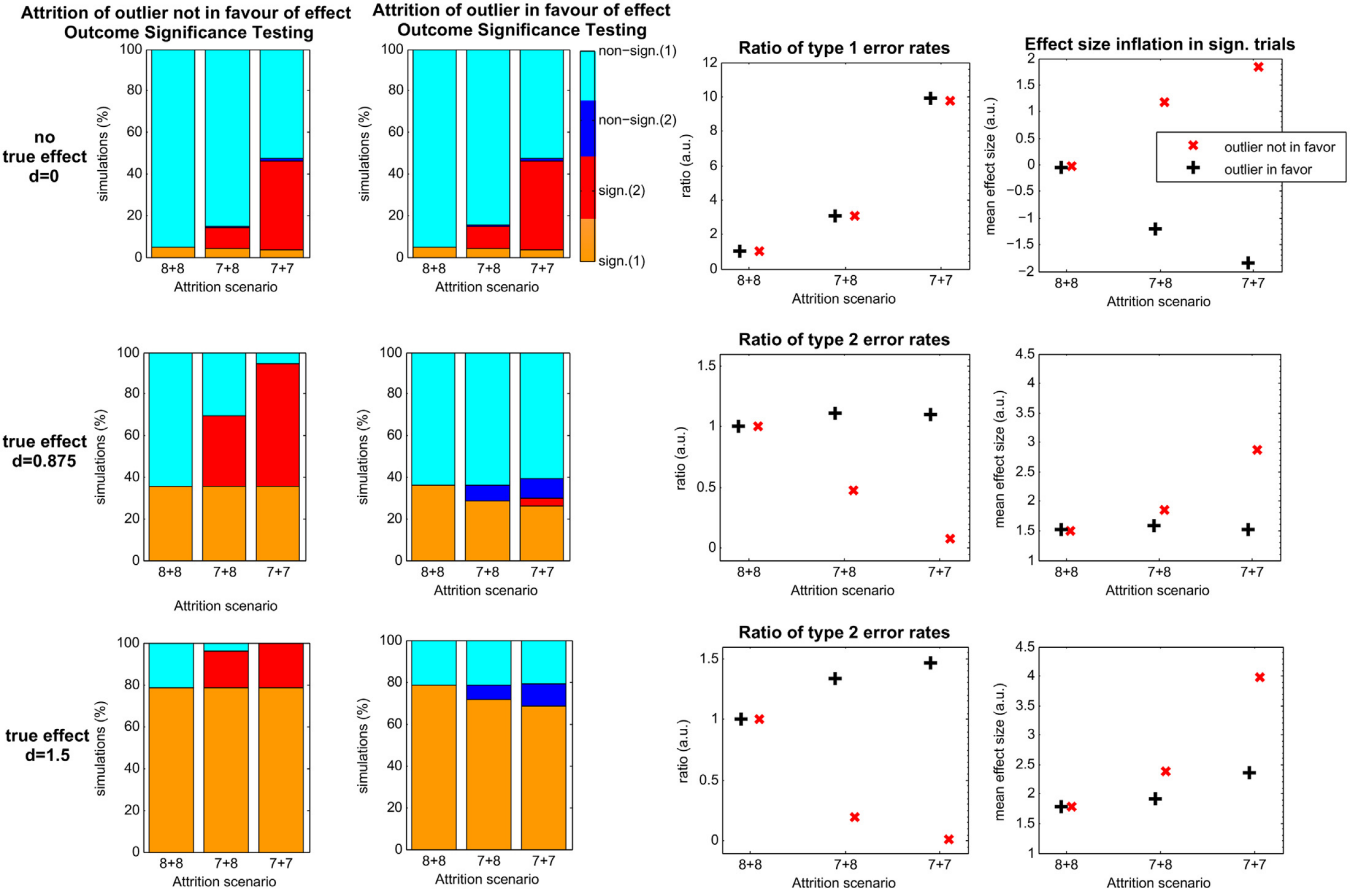
Simulation results for attrition of outliers. Column 1 + 2: rows represent the results of a different effect size (d) scenario, indicated left. The level of attrition in either treatment group is stated as “8 + 8” without attrition and “7 + 8” and “7 + 7” with the number of missing animals increasing from left to right. Probability of positive trials before and after attrition of outliers in the samples that are not in favor (first column) or in favor (second column) of the expected effect. Colors represent the proportion of trials out of 10,000 simulations that are significant (1) independent of attrition (orange) or significant (2) only in the case of attrition (red), non-significant (1) independent of attrition (cyan), or non-significant (2) only in the case of attrition (dark blue). Column 3: ratio of type 1 error rates (falsely accepting the H_1_ hypothesis if there is no true effect, first row) or type 2 error rates (falsely failing to reject the null hypothesis if there is a true effect, second and third row), respectively, with increasing attrition relative to the rates acquired with the full sample (“8 + 8”). Ratios for attrition of outliers that are in favor of the effect are colored in black, and ratios for attrition of outlier that are not in favor of the effect are colored in red, in arbitrary units (a.u.).

To explore the effects of attrition in preclinical studies, we simulated data for a two-armed trial (e.g., treatment versus control) beginning with a sample size of eight animals per group, a typical size reported in preclinical research [21,22]. We used scenarios with different true standardized effect sizes: d = 0 (no true difference between groups), d = 0.875 (a common preclinical reported effect size, [22]) and d = 1.5 (a strong effect for which 8 versus 8 is adequately powered). We simulated two forms of attrition: random loss (i.e., elimination of animals irrespective of group or outcome value) and biased removal (i.e., elimination of animal data points that undermine the expected effect). Biased removal of samples maximized the difference between experimental groups, irrespective of group membership (treatment versus control). Figs 1 and 2 show the probability of declaring statistically significant differences between simulation group means. The level of attrition in both treatment groups is stated as “8 + 8” without attrition and “7 + 8” to “6 + 7” as the total number of animals used decreases from left to right.

In the first set of simulations (Fig 1, row 1), we assumed there was no real effect (Cohen’s effect size d = 0, top row). Under these conditions, random attrition (first column) did not alter the false discovery rate of 5%. The effects of non-random attrition are reflected in the second column. Here, with removal of three samples disfavoring an expected effect (third column, attrition scenario “6 + 7”), the proportion of trials declaring statistically significant trials increased from 5% to 23%.

Though no true effect was present (d = 0), we also examined the impact of attrition on effect size estimates from statistically significant results. For significant experiments, (fourth column, first row), we observed a small increase in estimated effect size when three animals were randomly excluded d_est ‘6+7’_ = 0.09 (SE: 0.64). For non-random attrition, the estimated effect sizes were strikingly larger (e.g., d_est ‘6+7,’_ = 1.67 [SE: 0.66]).

In our second set of simulations (Fig 1, row 2), we simulated a preclinical study with a commonly observed effect size of Cohen’s d = 0.875 [21,22]. Here we see that with random loss of animals the risk for type 2 error decreased from 63% to 20%. However, this apparent advantage is offset by the loss in power and corresponding increase in false negative rate (from 63% without attrition to 73% for a loss of three animals, attrition scenario “6 + 7”). Biased removal of animals led to an artificial increase in the true positive rate from 37% without attrition to about 80% in the last scenario (“6 + 7”). Even with a true effect size of 0.875, the mean estimated effect size of significant trials was d_est’8+8’_ = 1.52 (SE: 0.57), an inflation of 175%. With attrition, this further increased to d_est’6+7’_ = 1.73 (SE: 0.66) with random attrition and to d_est’6+7’_ = 2.17 (SE: 0.71) with non-random attrition of three samples, corresponding to a striking 197% and 248% increase of the true effect size, respectively. These results follow from an increase in false negative rates due to the loss in power, in which only large effects can be detected. More information on the overestimation of effect size estimates for all attrition scenarios may be found in S1 Fig of the supporting information.

The third set of simulations (Fig 1, row 3) showed similar effects of attrition when we assumed a large true effect size (Cohen’s d = 1.5). Again, random loss decreased power and increased the false negative rate, accompanied by an inflated average effect size estimate among the significant experiments (d_est’6+7’_ = 1.97, SE: 0.69). Biased removal artificially increased the true positive rate from 79% to 98% when three samples were selectively dropped, with a corresponding decrease in type 2 error from 21% to 2%. The mean estimated effect size from significant trials was d_est’6+7’_ = 2.79 (SE: 0.80), which corresponded to 186% of the effect size in the total body of simulated studies.

In addition to attrition due to reasons such as illness or data loss, researchers often exclude measurements with extreme values (outliers). We therefore simulated the effects of removing outliers with random loss or biased removal. As expected, the impact of excluding outliers depended on whether the removed outliers were supportive of the expected effect (Fig 2, second column) or not (Fig 2, first column). When no effect is present (row 1), removal of outliers resulted in changes in effect sizes, especially when a low extreme value was removed from one group, and a high extreme from the other. Here, the false positive rate rose from 4.7% to 46%. In addition to striking type 1 error, estimated effect sizes from trials where d = 0 (no effect) were as much as d_est’7+7’_ = 1.85 (SE: 0.66). If a true effect was present (Fig 2, second and third row), attrition of outliers that opposed the effect simultaneously increased the true positive rate and decreased the risk for type 2 error from 63% to 5% and from 21% to almost 0% for an effect size of 0.875 or 1.5, respectively. The estimation of these effect sizes for positive trials inflated to d_est’7+7’_ = 2.88 (SE: 0.79) and d_est’7+7’_ = 3.98 (SE: 0.96), respectively. In contrast, attrition of outliers that formerly supported an effect decreased the true positive rate from 36% to 30% or from 79% to 69% for an underlying effect size of 0.875 or 1.5, respectively. Type 2 error also increased from 64% to 70% and from 21% to 31% (a risk ratio of 1.1 and 1.5).

Finally, we also simulated the effects of attrition on groups with larger sample sizes of 12, 16, 20, 24, and 30 animals (for details, see S1 Text). First, we explored the effects of losing three animals (or most severe scenario, above) in a random or targeted fashion in these larger groups. Here, the proportion of falsely significant trials decreased as sample sizes increased (S2 and S3 Figs), following from an increase in power. However, when a constant proportion (20%) of animals was removed from each comparison, larger group sizes could not protect against overestimation of effect size (S4 and S5 Figs).

By and large, the results of our simulation not only show that random exclusion of animals decreases the sample size and thus statistical power but also demonstrate that the exclusion of animals a targeted fashion, including removal of outliers, can have extreme consequences with regard to false positives and skewed interpretation.

## Meta-analysis of Preclinical Studies

To complement the results from our simulation study, we estimated the frequency and impact of attrition in a series of recent preclinical studies in cancer and stroke. Our meta-analyses employed two pre-existing datasets that have been described in detail elsewhere: Collaborative Approach to Meta-Analysis and Review of Animal Data from Experimental Studies (CAMARADES) [21,22] and Studies of Translation, Ethics and Medicine (STREAM) (S2 Text). Our search returned 100 papers on the topic of stroke and cancer, containing 316 experiments on infarct volume and 206 experiments on tumor shrinkage, respectively. To assess the presence of attrition, we compared reported numbers of animals in the methods and results section for each experiment. Experiments for which these numbers were reported as identical were coded as “Matched” (although unreported losses or exclusions cannot be completely ruled out), and experiments for which these numbers differed were coded as “Attrition.” Experiments for which this comparison was not possible were coded as “Unclear” (for more details, please see S1 Text).

**Fig 3:**
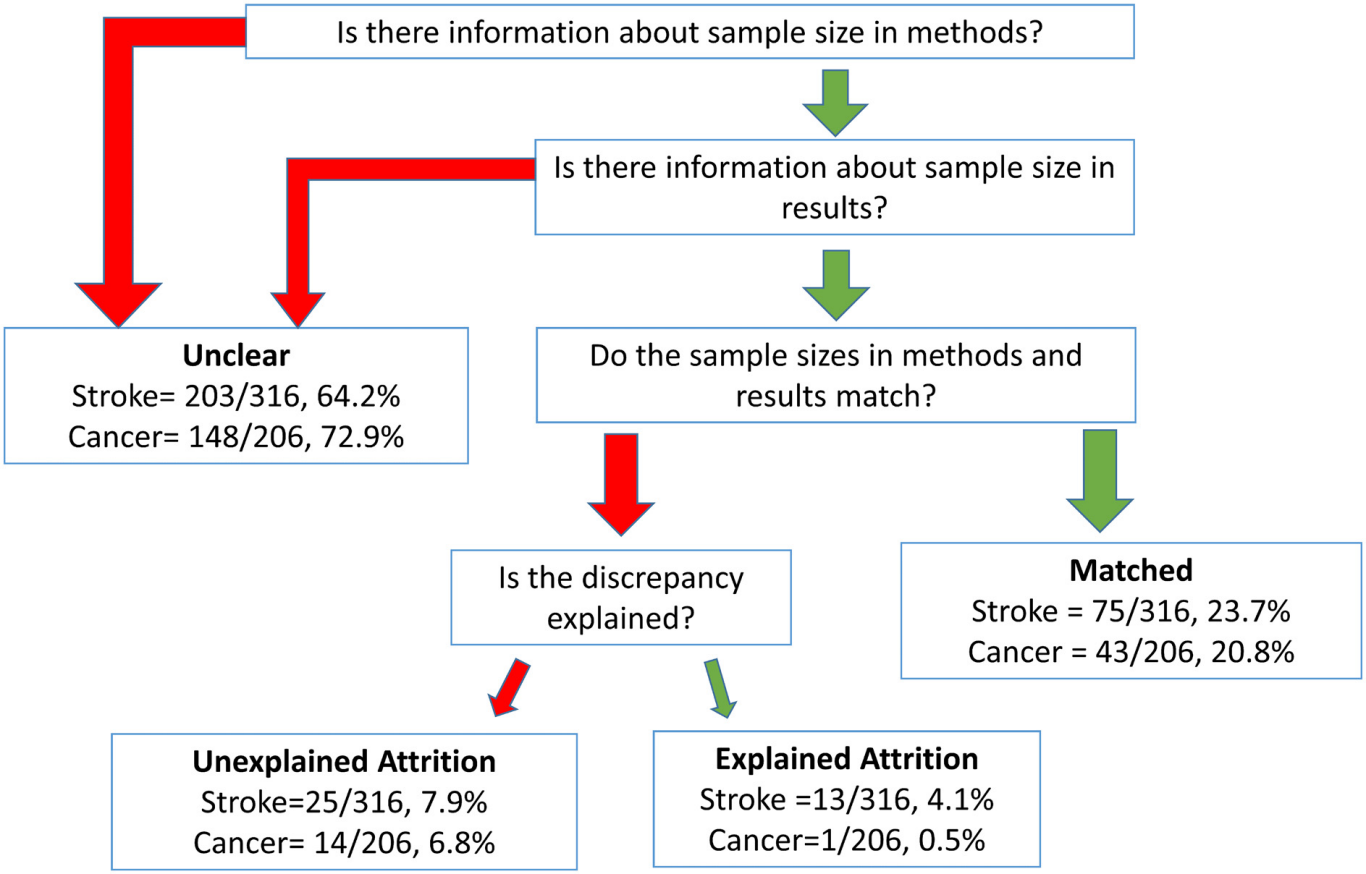
Procedure from meta-analysis for classifying papers based on type of animal reporting employed. All experiments were compared using the numbers of animals reported in the methods and results section. If this comparison was impossible, experiments were coded as “Unclear.” If these numbers were identical, experiments were coded as “Matched.” If there was a discrepancy between these numbers, then experiments were coded as “Attrition,” which could either be explained (via information in text or figure legends of the paper) or unexplained.

In both indication areas, animal numbers in more than half of the experiments had “Unclear” animal numbers, followed by those categorized as “Matched” and a small number with reported “Attrition” (see Fig 3). Within the category of “Attrition,” we differentiated between explained (exact numbers of animals lost and reasons given) and unexplained forms of animal loss. Among studies with documented attrition, numbers of missing animals were only explained in a small proportion of experiments (1/15 in cancer and 13/38 in stroke). To test whether papers with detected attrition were not exceptionally detailed reports, we checked all publications against a simple rubric of reporting quality concerning blinding practices. There was a significant difference between types of animal flow reporting and presence of blinding practices, indicated by a larger proportion of blinding reporting in the “Attrition” category, as well as a larger proportion without reporting of blinding in the “Unclear” group (Fisher’s exact: *p* < 0.001, S6 Fig).

**Fig 4:**
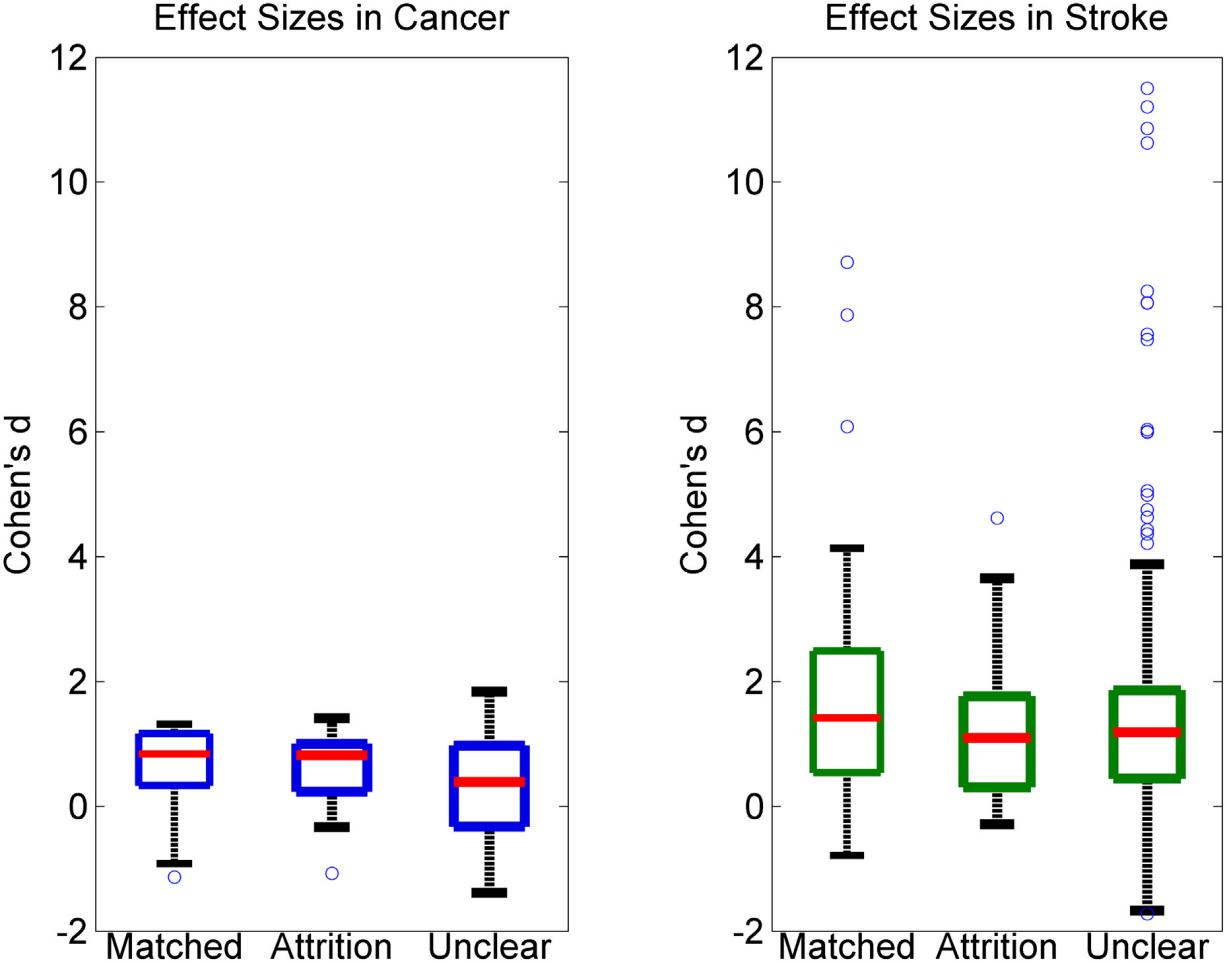
Effect sizes in experiments with different forms of animal reporting in stroke (left) and cancer (right). Boxes represent second to third quartiles, and red lines in the middle are the median. Whiskers represent first and fourth quartiles.

To see whether differences in reporting were associated with experimental effect size, we compared effect sizes across experiments coded as “Unclear,” “Matched,” and “Attrition.” In our sample, the vast majority of studies (152/206 in cancer, 276/316 in stroke) reported a “desired” effect, i.e., a better outcome for animals in the treatment group. All effect sizes, regardless of direction, were used in comparison between groups. In both stroke and cancer, “Matched” experiments displayed the highest median effect sizes (cancer: median d = 0.84, stroke: median d = 1.42 see Fig 4). Experiments coded as “Attrition” produced medium effect sizes in cancer (median d = 0.82) and the lowest median effect sizes in stroke (median d = 1.10). Finally, papers that were coded as “Unclear” reported the lowest median effect size in cancer (median d = 0.39) and an intermediate value in stroke (median d = 1.19). We identified a significant association between effect size and category of experimental reporting for cancer (Χ^2^(df = 2, *n* = 206) = 7.62, *p* = 0.022) but not for stroke (Χ^2^(df = 2, *n* = 316) = 2.70, *p* = 0.259). Within experiments that contained attrition, those with unexplained attrition had higher median effect sizes (median d = 0.97 interquartile range [IQR] [0.33–1.73]) than experiments in which the attrition was accounted for in the text (median d = 0.67 IQR [0.05–1.25]). This difference, however, was not significant in our data sample pooled across cancer and stroke (*p* = 0.343).

To check if our simulation study used realistic scenarios of animal loss (1/16–3/16 animals, i.e., 6.25%–18.75% loss), we examined what proportion of animals were lost experiments with detectable attrition. Almost half (47.1%, or 25/53) of experiments with attrition reported 25% animal loss or more. This is equivalent to or greater than the proportion of animals in our “worst case” attrition scenario (i.e., 3/16 animals, or 18.75% loss, see Figs 1 and 2, S1 Table). As may be seen in Fig 1, this can lead to effect sizes inflated by 25% to 175% amongst experiments with statistically significant results.

Next, we looked for markers that could be indicative of unreported missing animals in our sample. Here, we examined the symmetry of group sizes, i.e., whether there are the same number of animals in control and treatment groups. Since an equal number of animals in the different groups is the most efficient use in order to optimize power, any difference in group size can be regarded as a proxy for attrition (either random or biased) or even as a post-hoc addition of animals to grow statistical power or significance. In total, 219 experiments or 42.0% of our datasets had uneven group sizes, with a higher proportion of experiments with smaller sample size in the treatment groups (58%) compared to control groups (95% CI: 52%–65%). When attrition was fully reported, 64.1% of experiments appear to have lost animals in the treatment group.

## Discussion

Through statistical modelling and meta-analysis, we have shown that the loss of a few animals, as may often occur in preclinical studies, can distort true effects. Random loss of animals increases the occurrence of false negatives due to a decrease in sample size and statistical power (Fig 1), already a problem in small sample studies [8]. However, biased removal (Fig 1), which can occur because of subconscious bias, leads to an even greater probability of false positive results, particularly in settings in which real effect sizes are subtle to nil (Fig 1) [12]. Here, the negative effects of loss of power are exacerbated by potential for selection and other biases, severely undermining statistical inference. Increasing group sizes, therefore, helped to diminish these effects (S2 Fig). Dropping outliers, a common practice in many laboratories, can also have substantial effects (Fig 2). Though the impact of outlier attrition on average effect of all experiments might be minimal (since only ~5% of normally distributed values have outliers), its effect on this group is disproportionally large. Results of attrition in all scenarios may be further compounded by the fact that many studies show a preponderance of preclinical publications reporting statistically significant effects [7,21]. This may reflect publication bias, whereby studies failing to show statistical significance are not published. Thus, publication of predominantly positive experiments with biased attrition magnifies the distortion of treatment effects even more.

Although not unexpected, the finding that non-random attrition can decrease the number of false negatives is also of interest (Fig 1). Our simulations showed that non-random attrition can artificially overestimate detected effects sizes, which leads to an artificial increase in power by effectively testing a bigger but biased effect and thus results in a decrease of false negatives. This decrease in type 2 errors might be perceived as a positive benefit, but it is just due to bias caused by non-random attrition. Because of the typical, low sample sizes in experimental research, most studies are highly underpowered even without attrition, and scientists are even more at serious risk of missing smaller, more subtle effects when attrition is present [6–8].

Ultimately, the impact of attrition is dependent on the total sample size of the experiment at hand. In our simulation, our starting point was a sample size of 8 + 8, which is representative of many published experiments [21,22]. We would like to stress that although an increase in sample size does help to counteract the impact of attrition to some extent (S2–S5 Figs), it is not a safeguard to this phenomenon, especially when the attrition is done in a biased fashion.

When attrition was reported in experiments in our meta-analysis, the loss was often more than 25% of subjects. Yet, as shown in our simulations, even more moderate loss can have serious consequences that are not significantly diminished when group sizes are larger (Figs 1 and 2, S3 and S4 Figs). For example, animals in the treatment group may die because of drug toxicity, especially if they are weakened because of a strong experimental intervention. Since these animals cannot be considered “treatment successes” in any form, this guarantees bias unless there is some way of adjusting data for toxicity-induced loss. An example of this phenomenon from the field of neurovascular medicine may be found in [23]. Indeed, more attrition in our sample occurred in treatment groups compared to control groups, and treatment groups were also unexpectedly smaller when animal use was “Matched” or “Unclear.”

The latter finding is worrisome but underlies a limitation in our data: verifiable presence of attrition was impossible to judge in roughly 50% and 75% of “Unclear” experiments in cancer and stroke, respectively (see Fig 3). Detection of attrition using comparison of reported numbers from methods and results is only effective when group sizes are reported completely (i.e., numbers instead of ranges) and when the methods section is not altered after an experiment is completed. Our criteria for declaring non-attrition were permissive: we cannot rule out the possibility that even in cases of “Matched” animal reporting, attrition may have occurred but the prospectively intended group sizes were never reported. Hidden attrition in “Matched” experiments could be one reason why median effect sizes were highest in this category (Fig 4).

Notwithstanding the limitations of our data, we can use the results of our simulations to extrapolate on the effects detected in our meta-analysis. Within our sample, 235 experiments in stroke and cancer, or 44.9% of the total, reported uneven group sizes suggestive of attrition. Median effect size in these experiments was 1.2 (IQR: 0.3–1.8). If we assume that there was a distortion of results due to attrition in half of these experiments (with effect sizes > 0, *n* = 199) resulting in an overestimation of effect sizes of 80%, the median of the true effect sizes of all 235 experiments would be 0.7 (IQR: 0.3–1.5) instead of 1.2.

Despite preliminary exploration of uneven group sizes in our sample, our conclusions about effects of attrition in published literature must remain limited. Without access to initial protocols and the ability to view deviations from them, using group asymmetries to uncover attrition remains strictly speculative. Therefore, the true burden of attrition in preclinical research remains unknown, and our results here are most likely an underestimation. Until transparent reporting becomes the rule, rather than the exception, we must instead focus on productive ways to deal with animal loss.

Attrition is also a problem in clinical trials [11,13]. However, one major difference from preclinical work is the presence of well-established standards for reporting patient flow (i.e., [14]) and imputing missing data points [24,25]. Neither are routinely practiced in preclinical research, although interest in strategies for dealing with missing data is growing [18,26,27]. Animal Research: Reporting of In Vivo Experiments (ARRIVE) reporting guidelines for animal research, for example, mandate monitoring animal flow over the course of an experiment.

Attrition of animals is often unforeseen and does not reflect willful bias. However, there are several simple steps that the scientific community can use to diminish inferential threats due to animal attrition. First, we recommend that authors prespecify inclusion and exclusion criteria, as well as reasons for exclusion of animals. For example, the use of flowcharts to track animals from initial allocation until analysis, with attrition noted, improves the transparency of preclinical reporting. An added benefit of this approach lies in the ability to track systemic issues with experimental design or harmful side effects of treatment. Journal referees can also encourage such practices by demanding them in study reports. Finally, many simple statistical tools used in medicine could be adopted to properly impute (and report) missing data [27,28]. Overall, compliance with ARRIVE guidelines will aid in most, if not all, of the issues inherent to missing data in preclinical research and help structure a better standard for animal use and reporting.

## Supporting Information

S1 Fig Change of effect size with attrition estimated from significant trials only. Left column: mean estimated effect sizes for random (black) and non-random attrition (red). Right column: overestimation in percent compared to the simulated “true” effect size d = 0.875 and d = 1.5 (corresponding to 100%), respectively.
(TIFF)

S2 Fig Simulation results for random and non-random attrition of three samples (one from control group, two from treatment group) in dependence of increasing sample size. Rows represents the results of a different effect size (d) scenario as indicated on the left. The number of samples after attrition in either treatment group is given on the bottom (e.g., “6 + 7”), with the total number of samples before attrition given in brackets (e.g., “(8 + 8)”). Column 1 + 2: probability of positive trials after random attrition (first column) or non-random attrition of extremes that are not in favor of the effect (second column) for different effect sizes (rows 1–3). Colors represent the proportion of trials out of 10,000 simulations that are significant (1) independent of attrition (orange) or significant (2) only in the case of attrition (red), non-significant (1) independent of attrition (cyan), or non-significant (2) only in the case of attrition (dark blue).
(TIFF)

S3 Fig Effect size estimated from positive trials only. Mean estimated effect sizes are displayed in black (+) for random attrition and in red (×) for non-random attrition, in arbitrary units (a.u.).
(TIFF)

S4 Fig Simulation results for random and non-random attrition of 20% of samples (about 12.5% in the control group and about 25% in the treatment group) in dependence of increasing sample size. Each row represents the results of a different effect size (d) scenario as indicated on the left. The number of samples after attrition in either treatment group is given on the bottom (e.g., “6 + 7”), with the total number of samples before attrition given in brackets (e.g., “(8 + 8)”). Column 1 + 2: probability of positive trials after random attrition (first column) or non-random attrition of extremes that are not in favor of the effect (second column) for different effect sizes (row 1–3). Colors represent the proportion of trials out of 10,000 simulations that are significant (1) independent of attrition (orange) or significant (2) only in the case of attrition (red), non-significant (1) independent of attrition (cyan), or non-significant (2) only in the case of attrition (dark blue).
(TIFF)

S5 Fig Effect size estimated from positive trials only. Mean estimated effect sizes are displayed in black (+) for random attrition and in red (×) for non-random attrition, in arbitrary units (a.u.).
(TIFF)

S6 Fig Proportion of experiments with different animal flow reporting noted as employing different blinding practices. Fisher’s exact X^2^ test revealed a significant difference between types of animal flow reporting and presence of blinding practices Χ^2^(df = 4 *n* = 522) = 19.935, *p* < 0.001.
(TIFF)

S1 Table Simulation scenarios. (DOCX)

S1 Text Materials and methods. (DOCX)

S2 Text Structure of STREAM Preclinical Cancer Database. (DOCX)

## Abbreviations

ARRIVE: Animal Research: Reporting of In Vivo Experiments
CAMARADES: Collaborative Approach to Meta-Analysis and Review of Animal Data from Experimental Studies
CONSORT: Consolidated Standards of Reporting Trials
H_1_ hypothesis: alternative hypothesis
IQR: interquartile range
STREAM: Studies of Translation, Ethics and Medicine
STROBE: Strengthening the Reporting of Observational Studies in Epidemiology
