# Supplementary figures and images for: Where Have All the Rodents Gone? The Effects of Attrition in Experimental Research on Cancer and Stroke
Source: PLoS Biol. 2016 Jan 4;14(1):e1002331. doi: 10.1371/journal.pbio.1002331 (PMC4699644; doi:10.1371/journal.pbio.1002331)

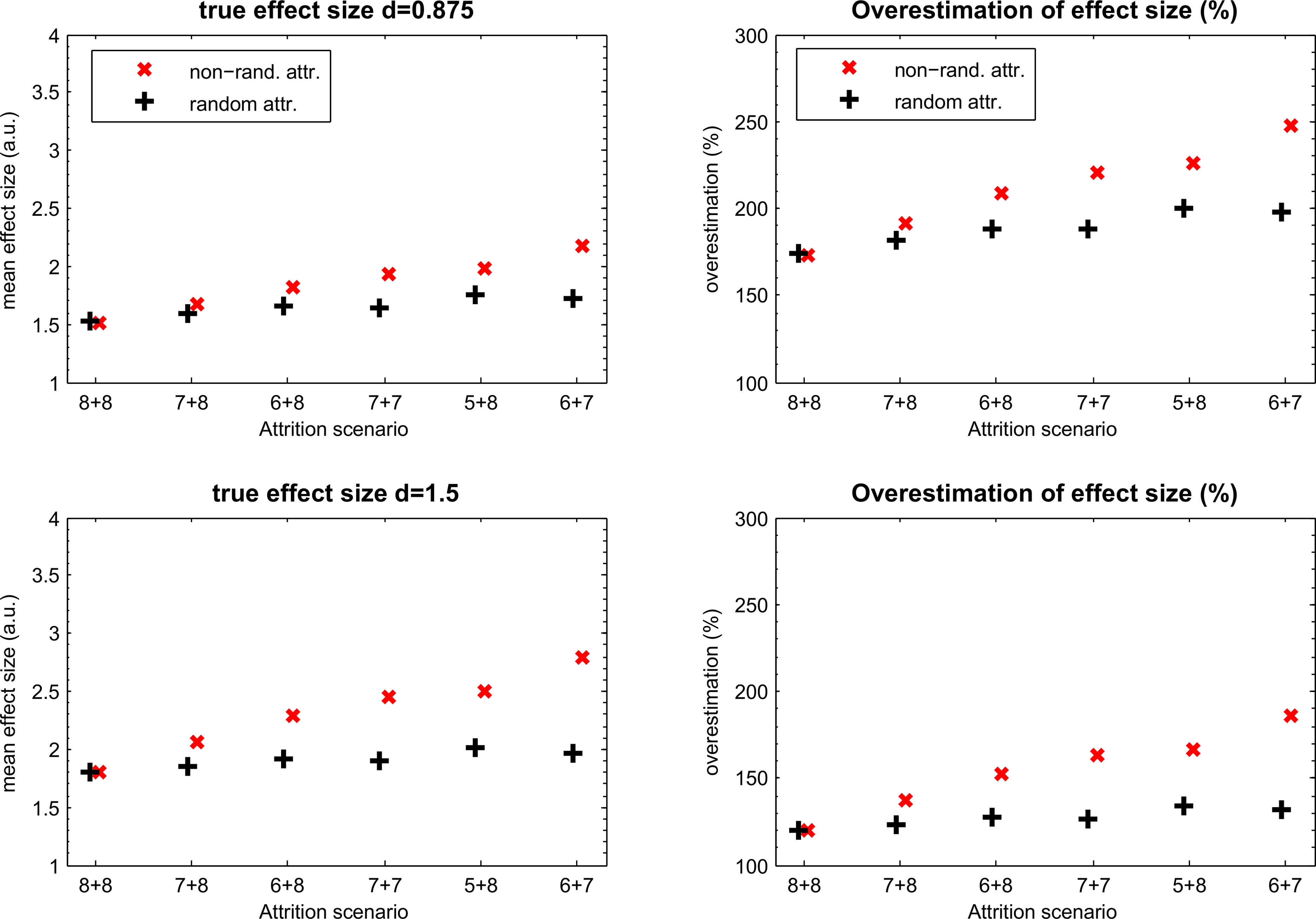

Supplement: S1 Fig — Left column: mean estimated effect sizes for random (black) and non-random attrition (red). Right column: overestimation in percent compared to the simulated “true” effect size d = 0.875 and d = 1.5 (corresponding to 100%), respectively. (TIFF) [file pbio.1002331.s001.tiff]

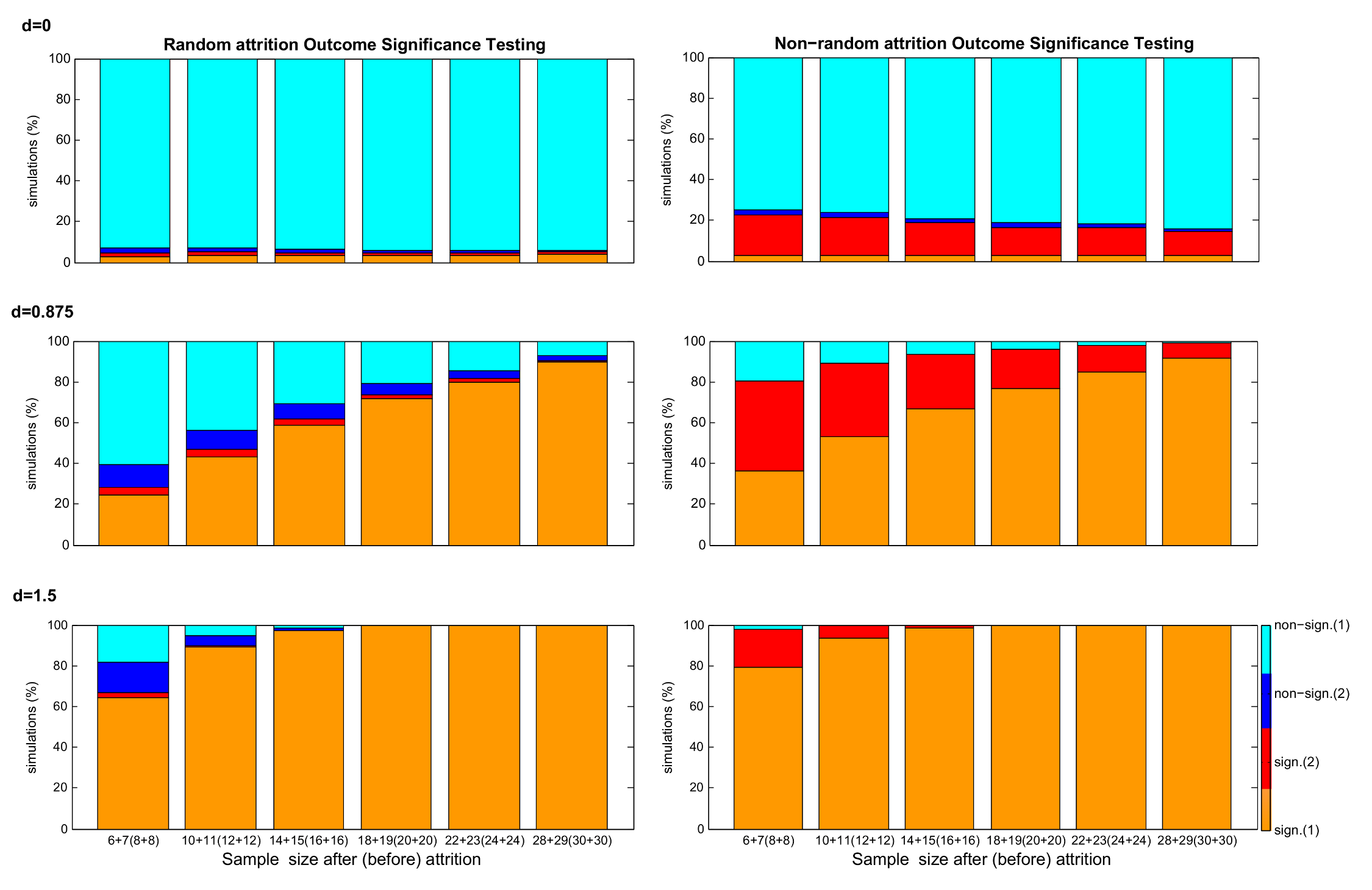

Supplement: S2 Fig — Rows represents the results of a different effect size (d) scenario as indicated on the left. The number of samples after attrition in either treatment group is given on the bottom (e.g., “6 + 7”), with the total number of samples before attrition given in brackets (e.g., “(8 + 8)”). Column 1 + 2: probability of positive trials after random attrition (first column) or non-random attrition of extremes that are not in favor of the effect (second column) for different effect sizes (rows 1–3). Colors represent the proportion of trials out of 10,000 simulations that are significant (1) independent of attrition (orange) or significant (2) only in the case of attrition (red), non-significant (1) independent of attrition (cyan), or non-significant (2) only in the case of attrition (dark blue). (TIFF) [file pbio.1002331.s002.tiff]

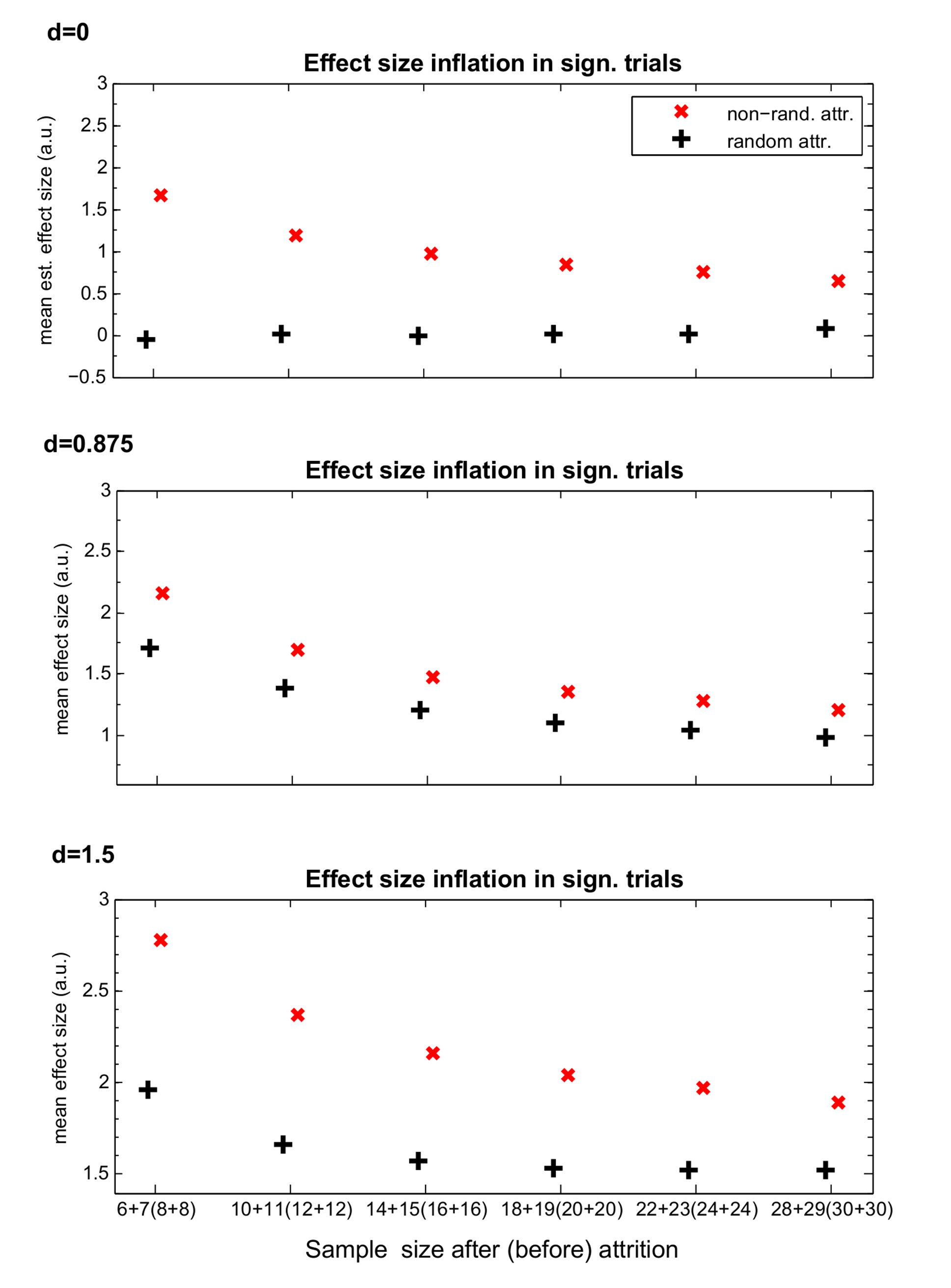

Supplement: S3 Fig — Mean estimated effect sizes are displayed in black (+) for random attrition and in red (×) for non-random attrition, in arbitrary units (a.u.). (TIFF) [file pbio.1002331.s003.tiff]

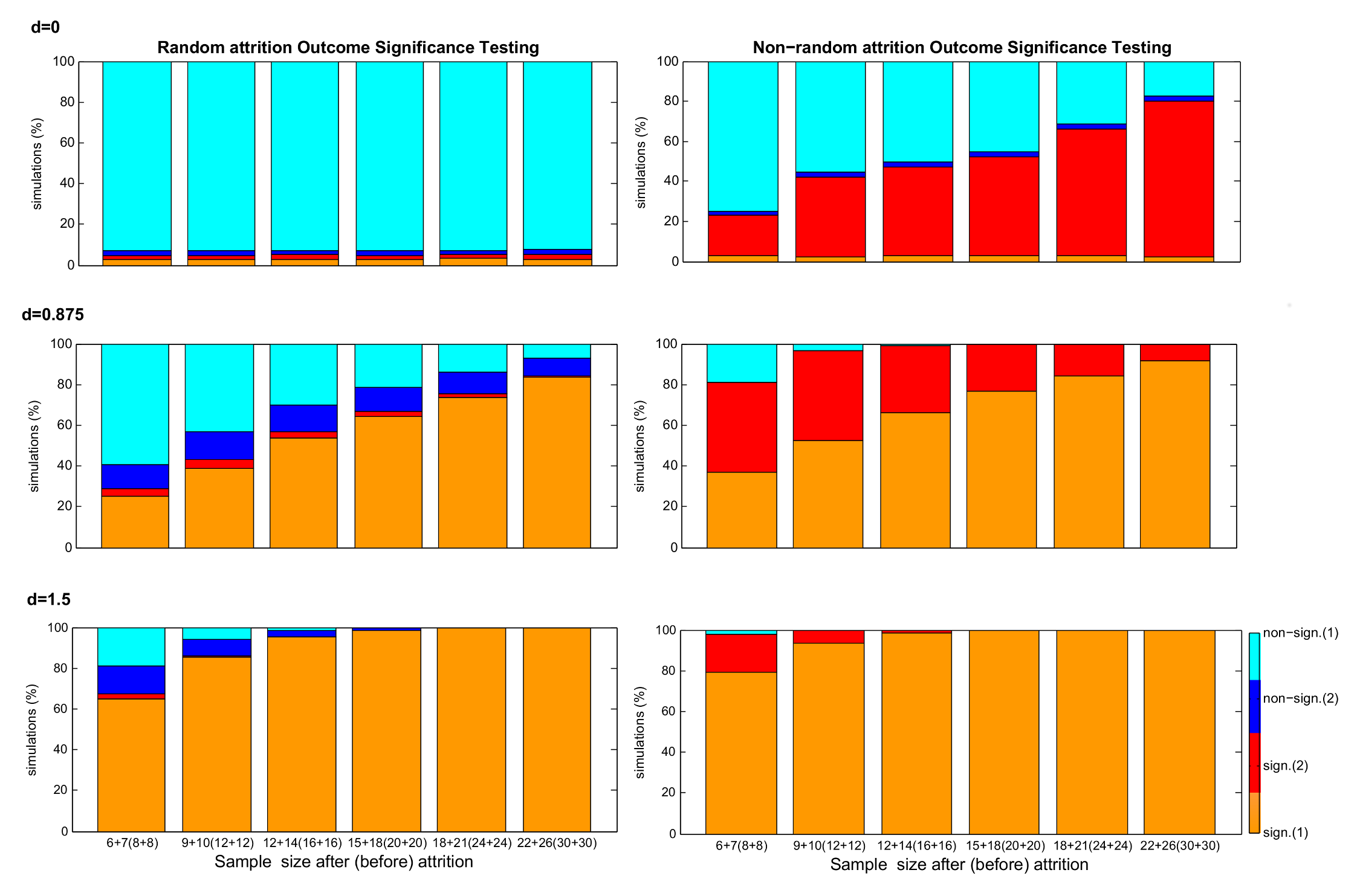

Supplement: S4 Fig — Each row represents the results of a different effect size (d) scenario as indicated on the left. The number of samples after attrition in either treatment group is given on the bottom (e.g., “6 + 7”), with the total number of samples before attrition given in brackets (e.g., “(8 + 8)”). Column 1 + 2: probability of positive trials after random attrition (first column) or non-random attrition of extremes that are not in favor of the effect (second column) for different effect sizes (row 1–3). Colors represent the proportion of trials out of 10,000 simulations that are significant (1) independent of attrition (orange) or significant (2) only in the case of attrition (red), non-significant (1) independent of attrition (cyan), or non-significant (2) only in the case of attrition (dark blue). (TIFF) [file pbio.1002331.s004.tiff]

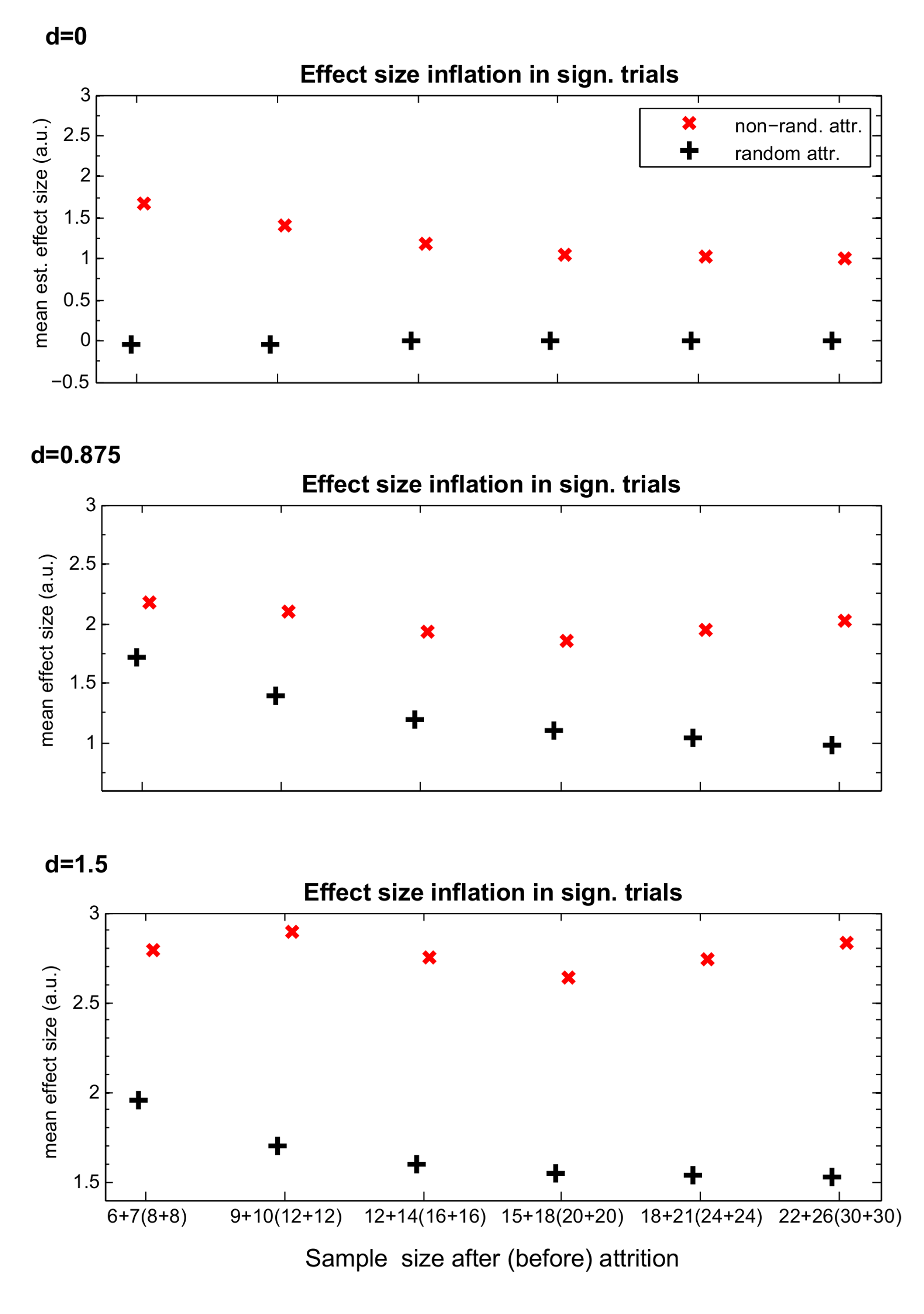

Supplement: S5 Fig — Mean estimated effect sizes are displayed in black (+) for random attrition and in red (×) for non-random attrition, in arbitrary units (a.u.). (TIFF) [file pbio.1002331.s005.tiff]

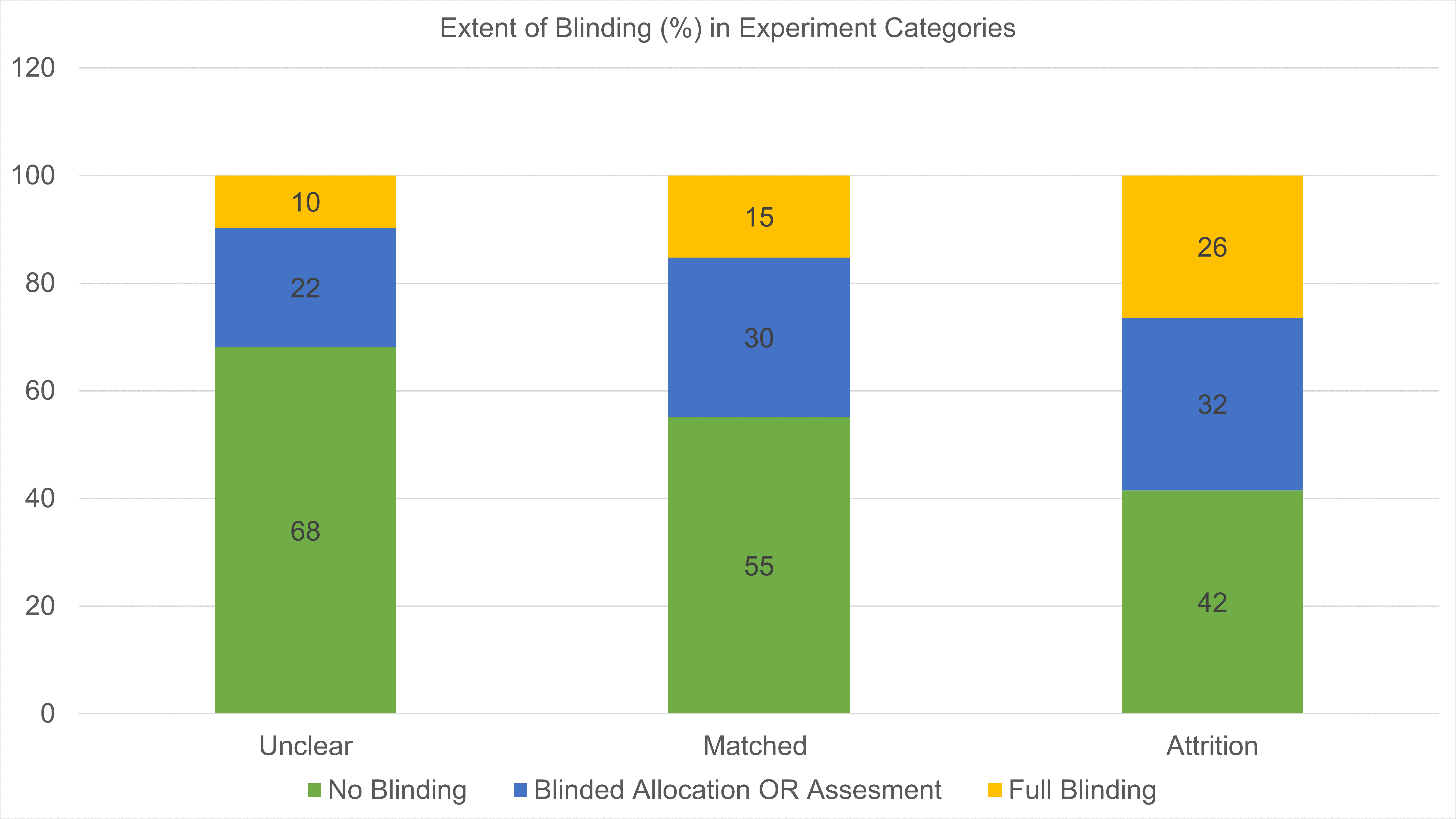

Supplement: S6 Fig — Fisher’s exact X2 test revealed a significant difference between types of animal flow reporting and presence of blinding practices Χ2(df = 4 n = 522) = 19.935, p < 0.001. (TIFF) [file pbio.1002331.s006.tiff]
