# Supplementary material for: Where Have All the Rodents Gone? The Effects of Attrition in Experimental Research on Cancer and Stroke
Source: PLoS Biol. 2016 Jan 4;14(1):e1002331. doi: 10.1371/journal.pbio.1002331 (PMC4699644; doi:10.1371/journal.pbio.1002331)
Supplement: S1 Table — (DOCX) [file pbio.1002331.s007.docx]

| **Scenario**  **Random/non-random attrition** | **Number of samples**  **Group 1 Group 2**  **(e.g. treatment) (e.g. controls)** | | **# total attrition** |
| --- | --- | --- | --- |
| **1** | 8 | 8 | 0 |
| **2** | 7 | 8 | 1 |
| **3** | 6 | 8 | 2 |
| **4** | 7 | 7 | 2 |
| **5** | 5 | 8 | 3 |
| **6** | 6 | 7 | 3 |
|  |  |  |  |
| **Scenario**  **Outlier attrition** | | | |
| **1** | 8 | 8 | 0 |
| **2** | 7 | 8 | 1 |
| **3** | 7 | 7 | 2 |
