# Supplementary material for: Where Have All the Rodents Gone? The Effects of Attrition in Experimental Research on Cancer and Stroke
Source: PLoS Biol. 2016 Jan 4;14(1):e1002331. doi: 10.1371/journal.pbio.1002331 (PMC4699644; doi:10.1371/journal.pbio.1002331)
Supplement: S1 Text — (DOCX) [file pbio.1002331.s008.docx]

**Supplementary Text S1: Material and Methods**

**Simulations**

For the main simulations in our study, data for a two-armed trial (e.g. treatment vs. control) with a typical sample size in current preclinical literature (N=8, [1]) was generated using the average values of studies collected in the CAMARADEs data base until December 2013. Based on the information provided by [2], the average effect size in preclinical research in mice is 0.875. Note that a study with 2x8 samples would have a power of only 37% to correctly detect this effect. However, with an effect size of 1.5, a study with the same sample size would have a power of 79% [3].

Samples were drawn from a normal distribution. Different simulation scenarios were set to result in three standardized effect sizes (Cohen’s d = ∆µ/σ, where d=1 signifies a difference in means between two groups equal to one standard deviation) of 0.0, 0.875 and 1.5. An independent t-test was performed for each data set, using a two-sided significance level of α=0.05. Simulations were performed with R software (version 3.2.1., [4]), using the MBESS package.

Three attrition scenarios were implemented: (1) random attrition, (2) attrition of animals disfavoring an experimental effect, and (3) attrition of outliers, defined as animals more than 2 standard deviations from the sample mean. This last scenario was repeated to model both the effects of removing animals that (i) strengthen and (ii) weaken the positive effect of the comparison. Each scenario was simulated 10,000 times. The proportions of significant trials for each attrition scenario are graphically displayed in **Fig 1** and **2**.

Additionally, we investigated the impact of random and selective attrition in larger samples. Here, we first simulated our most severe attrition scenario (loss of 3 animals) for several larger sample sizes with 12, 16, 20, 24 and 30 animals per group before attrition. We also simulated the effects of removing a constant proportion of animals (20%) from each of these groups. Results for different true standardized effect sizes (d=0, d=0.875 and d=1.5) are given in **S2** and **S3 Fig**.

For the outlier attrition scenarios we only used data sets with outliers. Attrition to undermine the positive effect of a comparison was performed by removing an outlier in group 1 higher than m1 + 2sd1 and the outlier in group 2 lower than m2 - 2sd2 (with µ1≤µ2). This was reversed to simulate attrition which boosts the positive effect of a comparison, or model observer/reporting bias. Either one or both of these outliers was removed. Table S1 gives an overview of all simulated scenarios.

Changes in type 1 error rates (the rate of falsely accepting the that there is a treatment effect, i.e. the proportion of significant trials for the simulations with effect size zero) and type 2 error rates (the rate of falsely failing to reject null hypothesis when there is a true effect, i.e. the proportion of non-significant results for the simulations with effect sizes 0.875 and 1.5) were calculated relative to the respective error rate without attrition.

Empirical effect sizes were estimated from the difference in group means divided by their pooled standard deviation. Here, only trials with a statistically significant effect were used, which inherently leads to an overestimation (inflation) of effect sizes [5]. We report mean estimated effect sizes with mean standard error over all significant trials for random and non-random attrition. They are also illustrated as relative change in percent compared to the a priori chosen simulated effect sizes of d=0.875 and d=1.5 of all trials in one scenario, respectively (see **S1 Fig**, right column).

**Meta-Analysis**

The meta-analyses used two pre-existing databases of preclinical studies: (1) The Collaborative Approach to Meta-Analysis and Review of Animal Data from Experimental Studies (CAMARADES) database of preclinical focal cerebral ischaemia studies [1, 2], and (2) The Studies of Translation, Ethics and Medicine (STREAM) database of preclinical experiments in cancer drugs [Supplementary Text S2]. From each, 100 papers were randomly selected. To be included in our study, these papers must have: been published between the years 2000 and 2013; contained at least one experiment quantifying infarct volume or tumor shrinkage, respectively; and provided data and variance for at least one treatment and control group. Only results of two standard measures- infarct volume and tumor shrinkage- were used in calculations from each paper.

We sorted experiments from each indication area according to whether they described attrition as occurring, whether attrition was not reported, and whether presence or absence of attrition was unclear. We classified studies as reporting attrition whenever numbers of animals reported in methods and results did not match. Experiments in which animal numbers did not change between methods and results were coded as “Matched”, although this designation cannot prove an absence of attrition- only that numbers in the publication were consistent. Papers were coded as containing “Attrition” if there was a discrepancy between these values, and the source of missing animals (i.e. treatment vs. control group) was noted. In many instances, however, it was impossible to directly compare animal numbers from the results and methods and these papers were placed under a third, “Unclear”, category. This usually stemmed from a failure to mention numbers in one of the sections, or mention them in a non-transparent way (i.e. “n>5 per group”). Furthermore, many papers reported animal numbers in a range of values (i.e. “n=7-12 per group”), rendering it impossible to directly compare group numbers in methods and results. As attrition can operate on the basis of observer bias, we additionally noted whether papers contained measures to promote blinded allocation and outcome analysis. All papers were additionally searched for any explanation of animal loss or attrition, i.e. declarations of surgical mortality or policy on exclusion of outlying values. This procedure is detailed in **Fig 3**. Every experiment was double-coded by two of the authors (CH and AD). Agreement between coders was 95.2%, and disagreements were resolved by consensus.

To assess the level of reporting detail for publications in our study, we used a rubric through which stroke and cancer papers could be directly compared. In this scale, and experiment scored ‘2’ if there was mention of both blinded allocation and assessment, ‘1’ if either one or the other approaches was mentioned, and ‘0’ if neither were. Thus, a ‘2’ indicates a high level of detail, and ‘0’ a low level.

Many studies in preclinical medicine begin with even group sizes that change over the course of the experiment. Apart from treatment cohorts that are almost always smaller (i.e. sham controls, not considered in this analysis), this may be indicative of animals lost, or added to analysis post-hoc. Therefore, to check whether there may have unmentioned attrition, we compared the sizes of the control and treatment groups in the results section of all experiments. If there was a discrepancy, the group with smaller numbers was noted.

Effect sizes for each experiment were calculated using Cohen’s d (see above, [6]), and statistically compared across groups and between papers with explained vs. unexplained attrition using Fisher’s exact Χ² test. To test if treatment groups were systematically smaller than control groups in case of different group sizes a binomial test was used, testing against the expected probability that the smaller group would be related to an intervention set at 50% (null hypothesis). All statistical calculations were performed using Matlab.

References:

1. Sena ES, van der Worp HB, Bath PM, Howells DW, Macleod MR. Publication bias in reports of animal stroke studies leads to major overstatement of efficacy. PLoS Biol. 2010; 8:e1000344.
2. CAMARDES. Review of animal data from experimental studies [Internet]. Edinburgh: CAMARDES; 2014 [cited May 14th, 2015]. Available from <http://www.dcn.ed.ac.uk/camarades/>.
3. Statistical Solutions. Nquery Advisor, version 7.0. 2007. Cork, Ireland.
4. Team, RC. R: a language and environment for statistical computing. Vienna, Austria: R Foundation for Statistical Computing; 2012. Open access available at: http://cran. r-project. org.

5. Colquhoun D. An investigation of the false discovery rate and the misinterpretation of p-values. R. Soc. Open sci. 2014; 1. doi:1098/rsos.140216.

1. Vesterinen H, Sena S, Egan K, Hirst T, Churolov L, Currie G et al. Meta-analysis of data from animal studies: A practical guide. J Neurosci Methods 2014;221:92-102.
