# Supplementary material for: Where Have All the Rodents Gone? The Effects of Attrition in Experimental Research on Cancer and Stroke
Source: PLoS Biol. 2016 Jan 4;14(1):e1002331. doi: 10.1371/journal.pbio.1002331 (PMC4699644; doi:10.1371/journal.pbio.1002331)
Supplement: S2 Text — (DOCX) [file pbio.1002331.s009.docx]

**Supplementary text S2: Structure of STREAM Preclinical Cancer Database:**

The STREAM database currently consists of three sections, containing papers collected for study of preclinical publications on different drugs in cancer research. The details of the collection and analysis of each section are explained below:

1. **Romidepsin**

Literature Search

We queried the following databases for in vivo, animal efficacy studies of romidepsin using a search strategy adapted from [1] and [2]: Ovid MEDLINE In-Process & Other Non-Indexed Citations and Ovid MEDLINE (dates of coverage from 1948 to 2012), EMBASE Classic and EMBASE database (dates of coverage from 1974 to 2012) and BIOSIS Previews (dates of coverage from 1969 to 2012). Search results were pooled into a single Endnote library and duplicates were removed. Additional citations were identified during screening and were added to the sample. Searches were performed on February 27, 2012.

Two reviewers independently performed title/abstract screening, and one individual performed full-text screening. Articles were screened on the following inclusion criteria: 1) original report of data (e.g. abstract, research article, or meta-analysis); 2) contain at least one experiment measuring cancer response in live, nonhuman animals and employing the agent; and 3) presented in English. If the same experiment was reported in different articles, the most detailed and/or recent publication was included. For details of exclusion criteria, please see PRISMA flowchart (available upon request).

Extraction

Each study was extracted at two levels: 1) “study-level” (e.g. whether the study contained a conflict of interest disclosure/presence, nature of clinical recommendations in discussion), and 2) “experiment-level” (e.g. how tumor growth curves were performed, and what their effect sizes were). All included studies were evaluated at the study-level, but only those with eligible experiments (defined as those evaluating the effect of romidepsin monotherapy on tumour volume or survival) were forwarded to experiment-level extractions where information on design and effect size was extracted.

Extraction parameters at the “experimental-level” were derived from a previously published systematic review of preclinical design guidelines, and were organized around three different validity types: internal (e.g. randomization, blinding); construct (e.g. choice and characterization of animal model); and external validity (e.g. replication of findings under varied conditions) (Henderson et al., 2013). We also extracted tumour growth and survival data (e.g. sample size, mean measures of control and treatment effects, and SDM/SEM) from romidepsin and untreated control arms using the “last common time point.” Extraction was performed by three independent coders using Distiller SR. There was a 20% full double-coding overlap and partial double-coding of seven targeted questions in all remaining articles (80%). Items targeted for complete double extraction were based on an inter-rater analysis showing less than 80% inter-rater agreement. Double-coding was performed in order to reduce inter-rater heterogeneity and discrepancies between coders were reconciled through discussion.

Meta-Analytic Methods

Effect sizes for tumor growth curves were calculated as Standardized Mean Differences (Cohen’s d) using Hedge’s g with 95% CIs. Pooled effect sizes were calculated using a random-effects model using the DerSimonian and Laird Method [3], in the OpenMeta Analyst software package for R [4]. In experiments containing greater than one dose of romidepsin, the SMD was pooled to create an aggregate SMD

1. **Sorafenib**

**Reference: James Mattina, Nathalie MacKinnon, Valerie Henderson, Dean Fergusson, and Jonathan Kimmelman. Design and Reporting of Targeted Anti-Cancer Preclinical Studies: A Meta-Analysis of Sorafenib *In preparation.***

Literature Search

Studies were identified by searching MEDLINE, BIOSIS and EMBASE databases on April 20, 2012 for trials using these search terms: “sorafenib,” or “Nexavar,” or variations on “BAY 43-9006” and MeSH terms including “preclinical,” “animals,” or search terms for commonly used animal models. The full search strategy, adapted from [1] and [2] is available upon request.

Study Selection

Inclusion criteria at the study level were a) primary data, b) full-text articles b) English language, c) investigated anti-cancer efficacy, d) measured a treatment effect in live, non-human animals, e) administered sorafenib as a control, comparator or treatment arm. For inclusion at the experiment level and quantitative meta-analysis, additional criteria were f) sorafenib monotherapy, g) evaluated drug effect on tumor volume, h) at least one common time measurement between control and treatment arms.

Data Extraction

We extracted experimental design elements derived from a prior systematic review of preclinical research guidelines [5]. These included at the study level: the names of the authors, the month and year of publication, the country associated with the corresponding author, the funding source(s), the conflict of interest statement, the molecular or physiological rationale for the experiment, the authors’ recommendation of sorafenib in the clinical setting as a monotherapy or combination. The design elements extracted at the experiment level included: sample size of each arm, treatment allocation, blinding of outcome assessment, inferential statistical test used, removal of animals throughout experiment, species, sex, weight, age, strain, immune status, disease modeled, disease stage, method of tumor initiation, transplantation site, transplantation size, transplant identity, drug administration schedule, administration method, days from disease induction to treatment, combination and comparator arms, day discontinuation of treatment, treatment effect at baseline, day 14 (or closest point), last time point and last common time point between control and treatment arms and SDM/SEM.

1. **Sunitinib**

**Reference: Valerie C. Henderson, Nadine Demko, Amanda Hakala, Nathalie MacKinnon, Carole A. Federico, Dean Fergusson, and Jonathan Kimmelman: Validity Threats and Clinical Correlates in Cancer Preclinical Research: A Meta-Analysis of Sunitinib. eLife** **4:e08351.**

***Literature Search***

To identify all *in vivo* animal studies testing the anti-cancer properties of sunitinib (“efficacy studies”), we queried the following databases on February 27, 2012 using a search strategy adapted from [1] and [2], available upon request: Ovid MEDLINE In-Process & Other Non-Indexed Citations and Ovid MEDLINE (dates of coverage from 1948 to 2012), EMBASE Classic and EMBASE database (dates of coverage from 1974 to 2012) and BIOSIS Previews (dates of coverage from 1969 to 2012). Search results were entered into an EndNote library and duplicates were removed. Additional citations were identified during the screening of identified articles. Screening was performed at citation level by two reviewers, and at full-text by one reviewer. Inclusion criteria were a) original reports or abstracts, b) English language, c) contained at least one experiment measuring disease response in a live, non-human animals, and d) employed sunitinib in a control, comparator, or experimental context, e) tested anti-cancer activity. In cases where the same experiment was reported in different articles, the most detailed and/or recent publication was included.

***Extraction***

All included studies were evaluated at the study-level, but only those with eligible experiments (e.g. those evaluating the effect of monotherapy on tumour volume) were forwarded to experiment-level extractions. Our primary outcome was tumour volume and we extracted necessary information (sample size, mean measure of treatment effect, and SDM/SEM) to enable calculation of study and aggregate level effect sizes. To account for different measures of tumour growth, Standardized Mean Differences (SMDs) using the last time point in common between the control and treatment arm after first sunitinib dose were calculated. Extraction was performed by four independent trained coders using DistilllerSR. There was a 12% double-coding overlap to 6 minimize inter-rater heterogeneity and prevent coder drift. Discrepancies in double coding were reconciled through discussion and, if necessary, by a third coder. The gross agreement rate for all double-coded studies was 83%.

**Supplementary Sources**

1. Hooijmans C, Tillema A, Leenaars M Ritskes-Hoitinga M. Enhancing search efficiency by means of a search filter for finding all studies on animal experimentation in PubMed. Lab Anim. 2010; 44: 170-5.

2. de Vries, R, Hooijmans C Tillema A, Leenaars M Ritskes-Hoitinga M. A search filter for increasing the retrieval of animal studies in Embase. Lab Anim. 2011; 45: 268-70.

1. DerSimonian, R. and N. Laird, Meta-analysis in clinical trials. Control Clin Trials. 1986; 7: 177-88.
2. Wallace B, Dahabreh I, Trikalinos T, Lau J, Trow P, Schmid C. Closing the gap between methodologists and end-users: R as a computational back-end. J Stat Softw. 2012; 49: 5.
3. Henderson V, Kimmelman J, Fergusson D, Grimshaw J, Hackam D. Threats to validity in the design and conduct of preclinical efficacy studies: A systematic review of guidelines for in vivoanimal experiments. PLoS Me 2013;10: e1001489.
